# Response to “Environmental scan and evaluation of best practices for online systematic review resources”

**DOI:** 10.5195/jmla.2018.496

**Published:** 2018-10-01

**Authors:** Chris Champion

**Affiliations:** Head of Membership, Learning and Support Services, Cochrane Central Executive, London, United Kingdom

**To the editor**, Cochrane staff read the recently published article, “Environmental Scan and Evaluation of Best Practices for Online Systematic Review Resources,” which is a helpful evaluation of online training resources for systematic review methodology, and noted that it included an assessment of the former Online Learning Modules for Cochrane Authors. Cochrane would like to make *Journal of the Medical Library Association* readers aware that those learning modules have been discontinued, and, in September 2017, Cochrane launched Cochrane Interactive Learning, a new, contemporary, and fully updated set of nine learning modules on conducting an intervention review.

The new Cochrane Interactive Learning provides more than twelve hours of self-directed learning on the complete systematic review process for both new and experienced review authors. It represents substantially more than just a change of name. Cochrane recognized that the former modules were out of date and did not reflect best contemporary online learning practice. The new resource was commissioned to address that.

The evaluation of resources in the published article was based on their content, design, interactivity, and usability. In the development of Cochrane Interactive Learning, staff carried out extensive research in relation to all of these elements to inform product development:

- Content: Cochrane Interactive Learning was developed in close collaboration with experts from Cochrane Methods Groups. It covers framing a review; searching for studies, risk-of-bias assessment, meta-analysis, GRADE, and Summary of Findings tables; integrating economic evidence; drawing conclusions; and reporting the review.
- Design: Each of nine Cochrane Interactive Learning modules is built from multiple topics so that learners can build knowledge one step at a time and focus on elements of review writing that they are less confident in.
- Interactivity: A variety of formats are used, including animations, video and audio recordings, interactive exercises, worked-out examples, and formal assessments with downloadable certificates on completion. As they work with the modules, learners can filter content, mark favorite pages, and monitor their own progress.
- Usability: Layout and navigation of Cochrane Interactive Learning was based on extensive user testing and reflects best practice in modern interactive learning design, accessibility, and pedagogy. The modules are designed to give access to learning at the point of need, working on most devices, including laptops, tablets, and mobile phones.

Based on the feedback that we have received from institutions, we are confident that those engaged in supporting systematic review writing in their institutions will be interested in making this new resource available to their local research community in the same way that Cochrane has made it available to all Cochrane authors.

